# Risk prediction model for mortality in microscopic polyangiitis: multicentre REVEAL cohort study

**DOI:** 10.1186/s13075-023-03210-8

**Published:** 2023-11-20

**Authors:** Takuya Kotani, Shogo Matsuda, Ayana Okazaki, Daisuke Nishioka, Ryu Watanabe, Takaho Gon, Atsushi Manabe, Mikihito Shoji, Keiichiro Kadoba, Ryosuke Hiwa, Wataru Yamamoto, Motomu Hashimoto, Tohru Takeuchi

**Affiliations:** 1 Department of Internal Medicine (IV), Division of Rheumatology, Osaka Medical and Pharmaceutical University, Daigaku-Machi 2-7, Takatsuki, Osaka 569-8686 Japan; 2 Department of Medical Statistics, Research & Development Center, Osaka Medical and Pharmaceutical University, Osaka, Japan; 3 Department of Clinical Immunology, Osaka Metropolitan University Graduate School of Medicine, Osaka, Japan; 4 Department of Rheumatology and Clinical Immunology, Kyoto University Graduate School of Medicine, Kyoto, Japan; 5 Department of Health Information Management, Kurashiki Sweet Hospital, Kurashiki, Japan

**Keywords:** Microscopic polyangiitis, Prognosis, Risk prediction model, Multicentre cohort study

## Abstract

**Background:**

To establish refined risk prediction models for mortality in patients with microscopic polyangiitis (MPA) by using comprehensive clinical characteristics.

**Methods:**

Data from the multicentre Japanese registry of patients with vasculitis (REVEAL cohort) were used in our analysis. In total, 194 patients with newly diagnosed MPA were included, and baseline demographic, clinical, laboratory, and treatment details were collected. Univariate and multivariate analyses were conducted to identify the significant risk factors predictive of mortality.

**Results:**

Over a median follow-up of 202.5 (84–352) weeks, 60 (30.9%) of 194 patients died. The causes of death included MPA-related vasculitis (18.3%), infection (50.0%), and others (31.7%). Deceased patients were older (median age 76.2 years) than survivors (72.3 years) (*P* < 0.0001). The death group had shorter observation periods (median 128.5 [35.3–248] weeks) than the survivor group (229 [112–392] weeks). Compared to survivors, the death group exhibited a higher smoking index, lower serum albumin levels, higher serum C-reactive protein levels, higher Birmingham Vasculitis Activity Score (BVAS), higher Five-Factor Score, and a more severe European Vasculitis Study Group (EUVAS) categorization system. Multivariate analysis revealed that higher BVAS and severe EUVAS independently predicted mortality. Kaplan–Meier survival curves demonstrated lower survival rates for BVAS ≥20 and severe EUVAS, and a risk prediction model (RPM) based on these stratified patients into low, moderate, and high-risk mortality groups.

**Conclusions:**

The developed RPM is promising to predict mortality in patients with MPA and provides clinicians with a valuable tool for risk assessment and informed clinical decision-making.

**Supplementary Information:**

The online version contains supplementary material available at 10.1186/s13075-023-03210-8.

## Background

Microscopic polyangiitis (MPA) is a subset of anti-neutrophil cytoplasmic antibody (ANCA)-associated vasculitis (AAV) characterized by small-vessel inflammation that affects organs such as the skin, lungs, and kidneys [1]. Despite medical advancements, the determinants of survival in patients with MPA remain unclear. Prior research has clarified aspects of the overall survival and causes of death in patients with MPA, revealing a wide spectrum of outcomes [2–5]. Cumulative survival rates have been reported in systematic reviews at distinct time intervals, fluctuating between 13 and 67% for the overall mortality, 77 and 100% for 1-year survival, 46 and 85% for 5-year survival, and 60 and 80% for 10-year survival [2]. Nevertheless, a substantial number of the deaths are due to vasculitis and immunosuppressive therapy [2, 6], underscoring the necessity for a comprehensive exploration of adverse prognostic factors that contribute to mortality in MPA.

Although various studies have identified prognostic indicators linked to MPA, such as age, renal involvement, and treatment modalities [2], the precise predictors of mortality in patients with MPA remain unclear. Recent studies have shed light on potential factors correlating with increased mortality risk, such as advanced age, elevated serum creatinine levels, reduced albumin levels, and lung involvement, including diffuse alveolar haemorrhage and/or interstitial lung disease [7–11]. These findings underscore the intricate nature of MPA’s clinical course and emphasize the importance of gaining a more nuanced understanding of its prognostic determinants○.

Motivated by the knowledge gap in the negative prognostic factors linked to death in MPA, we aimed to construct a comprehensive predictive model for mortality among patients with MPA. To this end, we utilized the data from a multicentre cohort study of vasculitis in Japan. We determined and validated strong predictors, which may improve the ability to estimate patient outcomes and guide clinical decisions. The findings of this study clarify the intricate interplay of clinical traits, disease manifestations, and patient demographics in MPA mortality. This study has the potential to markedly improve our understanding of the clinical trajectory of MPA, ultimately contributing to enhanced patient care and outcomes.

## Materials and methods

### Patients

This retrospective multicentre observational study was conducted using the Registry of Vasculitis Patients to Establish the REAL World Evidence (REVEAL) cohort to elucidate the prognostic factors associated with mortality and develop a predictive model for mortality in patients with MPA. The REVEAL cohort encompasses a multicentre observational registry of patients with MPA within the Kansai District of Japan. The dataset comprised information from three participating institutions: Osaka Medical and Pharmaceutical University, Kyoto University, and Osaka Metropolitan University. Between May 2005 and June 2021, 211 patients diagnosed with MPA according to the Chapel Hill Consensus definition [1] were enrolled. Upon enrolment, retrospective data were retrieved from electronic health records facilitated by a designated clinician at each centre. All patients underwent hospitalization for remission induction therapy, except for one patient who received immunosuppressive treatment at the physician’s discretion. Comprehensive clinical and laboratory data, treatment modalities, and outcomes were carefully extracted from the medical records. The REVEAL cohort was also used to assess the relapse and follow-up survival outcomes.

### Ethical considerations

This study adhered to the Declaration of Helsinki and its amendments. Approval was granted by the Ethics Committee of Osaka Medical and Pharmaceutical University and the Faculty of Medicine (Approval No. 1529), as well as the individual participating centres, namely Kyoto University (Approval No. R1540) and Osaka Metropolitan University (Approval No. 2021-074). The Ethics Committee of Kyoto University waived the need for patient informed consent given the anonymized nature of the data. Written informed consent was obtained from patients at other participating institutes.

### Clinical and laboratory assessment

The REVEAL cohort database served as the source for the extraction of various data points from the medical records, including demographic characteristics such as age at admission, sex, and smoking history (Brinkman index). Peripheral laboratory parameters recorded upon admission included white blood cell count, haemoglobin (Hb) levels, albumin, creatinine, C-reactive protein (CRP) levels, myeloperoxidase-specific anti-neutrophil cytoplasmic antibody (MPO-ANCA), and proteinase 3-specific anti-neutrophil cytoplasmic antibody (PR3-ANCA).

### Assessment of disease severity

Systemic disease activity was assessed using the BVAS, version 3 [12]. The European Vasculitis Study Group (EUVAS) categorization system [13] was adopted to classify disease severity. Additionally, each patient was evaluated using the 2009 Five-Factor Score (FFS), which was designed to predict outcomes at the time of MPA diagnosis [14].

### Statistical analysis

Data are presented as medians and interquartile ranges. Fisher’s exact test was employed as appropriate, while the Mann–Whitney U test was used to compare median values. Statistical significance was set at* P* < 0.05. Receiver operating characteristic (ROC) curve analysis was used to determine the optimal cut-off level to predict mortality among patients with MPA. The Kaplan–Meier method was utilized for survival curve assessment, and the significance of intergroup disparities was assessed using the log-rank test. Survival duration was calculated based on the time of remission induction therapy at each institution and the time of termination at the most recent hospital visit, or the date of censoring due to death. Comparisons of demographic and background characteristics were conducted between the two groups, and potential risk factors linked to mortality were extracted, with significance values of *P* < 0.05 in the univariate analysis. Hazard ratios for patient outcomes were estimated by univariate and multivariate analyses using a Cox regression model. In Cox proportional hazards models, age was treated as a covariate to account for potential confounding effects. Data analyses were performed using JMP version 17.0 (SAS Institute Inc., Cary, NC).

## Results

### Patient profiles

Among the 211 patients with MPA, 194 had new-onset MPA, and 17 were excluded since they were relapse cases. During a median follow-up period of 202.5 (84–352) weeks, 60 (30.9%) of the 194 patients died. The causes of death were MPA-related vasculitis in 11 (18.3%), infection in 30 (50.0%), and other causes in 19 (31.7%) patients; the details of mortality-causes are shown in Additional file 1. We compared the baseline clinical characteristics of surviving and deceased patients with MPA (Table 1). The median ages of the death and survival groups were 76.2 (71.5–83.1) and 72.3 (65.9–77.2) years, respectively, with significant difference (*P* < 0.0001). Furthermore, the median follow-up periods differed notably, with the death and survival groups being followed for 128.5 (35.3–248) and 229 (112–392) weeks, respectively. The smoking index was significantly higher in the death group at 950 (800–1200) compared to the survival group at 600 (300–920) (*P* = 0.002). Additionally, the death group exhibited significantly lower serum albumin levels at 2.5 (2.1–2.9) g/dL in comparison to the survival group’s level of 2.8 (2.3–3.3) g/dL (*P* = 0.025). Conversely, serum CRP levels were significantly higher in the death group than in the survival group, 9.3 (4.2–13.3) mg/dL vs 6.8 (1.5–12.0) mg/dL, respectively (*P* = 0.031). Concerning disease severity, BVAS was significantly higher in the death group than in the survival group, 17.5 (11.3–22.8) vs 12 (7.0–18.0), respectively (*P* = 0.005). The FFS was also significantly higher in the death group than in the survival group (*P* = 0.019). Moreover, the EUVAS categorization system also indicated significantly more severe cases in the death group than in the survival group (*P* = 0.037). Additionally, the death group had a significantly higher rate of plasma exchange than the survival group (*P* = 0.018).

**Table 1:** Baseline characteristics, initial treatments, and outcomes for 194 patients with MPA in the REVEAL cohort

Characteristics	Death (*N* = 60)	Survive (*N*= 134)	*P* value
Age, year	76.2 (71.5–83.1)	72.3 (65.9–77.2)	< 0.0001
Female,* n* (%)	29(48.3)	78 (58.2)	0.215
Observation period from baseline, weeks	128.5 (35.3–248)	229 (112–392)	< 0.0001
Smoking history, *n* (%)	31 (56.4)^a^	63 (51.6)^b^	0.627
Smoking index	950 (800–1200)	600 (300–920)	0.002
Laboratory findings			
WBC, /mm3	12005 (8063–16,648)	10110(7300–13,815)	0.086
Hb, g/dL	9.7 (8.2–11.9)	10.3 (9.0–11.9)^c^	0.325
Alb, g/dL	2.5 (2.1–2.9)	2.8 (2.3–3.3)^c^	0.025
Cre, mg/dL	1.34 (0.8–2.2)	1.0 (0.67–2.04)	0.056
CRP, mg/dL	9.3 (4.19–13.29)	6.8 (1.48–12.0)	0.031
Positive, anti-MPO-ANCA, *n* (%)	59(98.3)	133 (99.3)	0.524
Positive, anti-PR3-ANCA, *n* (%)	3 (5.0)	7 (5.2)	1
MPO-ANCA titre, U/mL	122.5 (57.7–240.8)	119 (46.1–251.0)	0.95
BVAS at onset	17.5 (11.3–22.8)	12 (7.0–18.0)	0.005
Five factor score 2009			
≤1	5 (8.3)	34 (25.4)	0.019
2	39 (65.0)	68 (50.8)	
≥3	16 (26.7)	32 (23.9)	
EUVAS categorization system			
Localized	1 (1.7)	6 (4.5)	0.037
Early systemic	12 (20.0)	34 (25.4)	
Systemic	30 (50.0)	78 (58.2)	
Severe	17 (28.3)	16 (11.9)	
Initial treatment			
PDN, mg/kg/day	1.0 (0.6–1.0)	1.0 (0.6–1.0)^c^	0.829
MPDN pulse therapy, *n* (%)	20 (35.1)	40 (32.0)	0.735
Immunosuppressants			
IVCY, *n* (%)	21 (37.5)	50 (42.7)	0.621
Total IVCY dose (g)	0.13 (0–1.0)	0.0 (0–2.0)	0.589
RTX, *n* (%)	4 (6.7)	12 (9.0)	0.78
IVIG, *n* (%)	1 (1.7)	7 (5.2)	0.439
AZA, *n* (%)	33 (55.0)	70 (52.2)	0.757
MMF, *n* (%)	1 (1.7)	2 (1.5)	1
Plasma exchange,* n* (%)	9 (15.0)	6 (4.5)	0.018
Haemodialysis, *n* (%)	7 (11.7)	10 (7.5)	0.411
Relapse, *n* (%)	22 (36.7)	50 (37.3)	1
Relapse, time	0 (0–1)	0 (0–1)	0.83
Cause of death, *n* (%)	MPA-related vasculitis 11 (18.3), infections 30 (50.0), others 19 (31.7)	-	-

*MPA* Microscopic polyangiitis, *WBC* White blood cell; *Hb* Haemoglobin, *Alb* Albumin *Cre* Creatinine, *CRP* C-reactive protein, *MPO-ANCA* Myeloperoxidase-anti-neutrophil cytoplasmic autoantibody, *PR3-ANCA* Proteinase 3-anti-neutrophil cytoplasmic antibody, *BVAS* Birmingham Vasculitis Activity Score, *EUVAS* European Vasculitis Study Group, *PDN* Prednisolone, *MPDN *Methylprednisolone, *IVCY* Intravenous cyclophosphamide, *RTX* Rituximab, *IVIG* Intravenous immunoglobulin, *AZA* Azathioprine, *MMF* Mycophenolate mofetil

The laboratory markers are presented as the median (interquartile range)

^a^Number of subjects, *n*= 55

^b^Number of subjects, *n*=122

^c^Number of subjects, *n*=133

### Cox regression analysis of deaths in MPA

The following were identified as risk factors: older age, higher smoking index, lower serum Alb levels, higher serum CRP levels, higher BVAS score at onset, higher FFS, and a more severe EUVAS categorization system. Next, we performed univariate analyses of these risk factors using a Cox regression analysis (Table 2); this model showed that older age, higher BVAS at onset, FFS 2, FFS ≥3 (vs FFS 0, 1), and severe EUVAS categorization (vs systemic) were predictors of death in MPA (*P* ≤ 0.001, <0.001, <0.001, 0.010, and 0.031, respectively). Since previous studies have shown that older age is associated with increased mortality in patients with MPA [2, 7, 15], we performed a multivariate Cox regression analysis to determine whether these predictors were independently associated with death after adjusting for age. This analysis revealed that a higher BVAS score and a severe disease according to the EUVAS categorization system were risk factors for death in MPA (*P* = 0.001 and 0.002, respectively).

**Table 2:** Prognostic factors of death in MPA patients

	Unadjusted			Age-adjusted		
Risk factors	Crude HR	95% CI	*P*	Adjusted HR	95% CI	*P*
Age (by year)	1.110	1.072–1.151	<0.001			
Smoking index	1.080	0.974–1.174	0.135			
Alb (g/dL)	1.001	0.999–1.001	0.054			
CRP (mg/dL)	1.038	0.995–1.083	0.084			
BVAS at onset	1.062	1.029–1.095	<0.001	1.054	1.021–1.087	0.001
Five-Factor Score 2009						
2 (ref; ≤1)	4.308	1.865–12.506	<0.001	2.054	0.799–5.277	0.135
≥3 (ref; ≤1)	3.578	1.339–11.217	0.010	1.541	0.538–4.412	0.42
≥3 (ref; 2)	0.830	0.428–1.507	0.5535			
EUVAS categorization system						
Early systemic (ref; localized)	2.932	0.575–53.477	0.23			
Systemic (ref; localized)	2.369	0.507–42.202	0.329			
Severe (ref; localized)	4.692	0.959–84.637	0.058			
Systemic (ref; early systemic)	0.808	0.423–1.644	0.54			
Severe (ref; early systemic)	1.600	0.765–3.452	0.213			
Severe (ref; systemic)	1.981	1.066–3.560	0.031	2.725	1.463–5.076	0.002

*HR* Hazard ratio, *CI* Confidence interval, *ref* Reference, *MPA*, Microscopic polyangiitis, *Alb* albumin, *CRP* C-reactive protein, *BVAS* Birmingham Vasculitis Activity Score, *EUVAS* European Vasculitis Study Group

The hazard ratios of death due to infection were derived from univariable and multivariable Cox regression analysis

### Cut-off values for BVAS at onset and survival curves

ROC curve analysis of baseline BVAS scores showed that a cut-off score of 19 maximized the AUC (0.627; sensitivity 43.3%; specificity 76.1%), indicating the best cut-off for predicting death (Additional file 2).

**Fig. 1:**
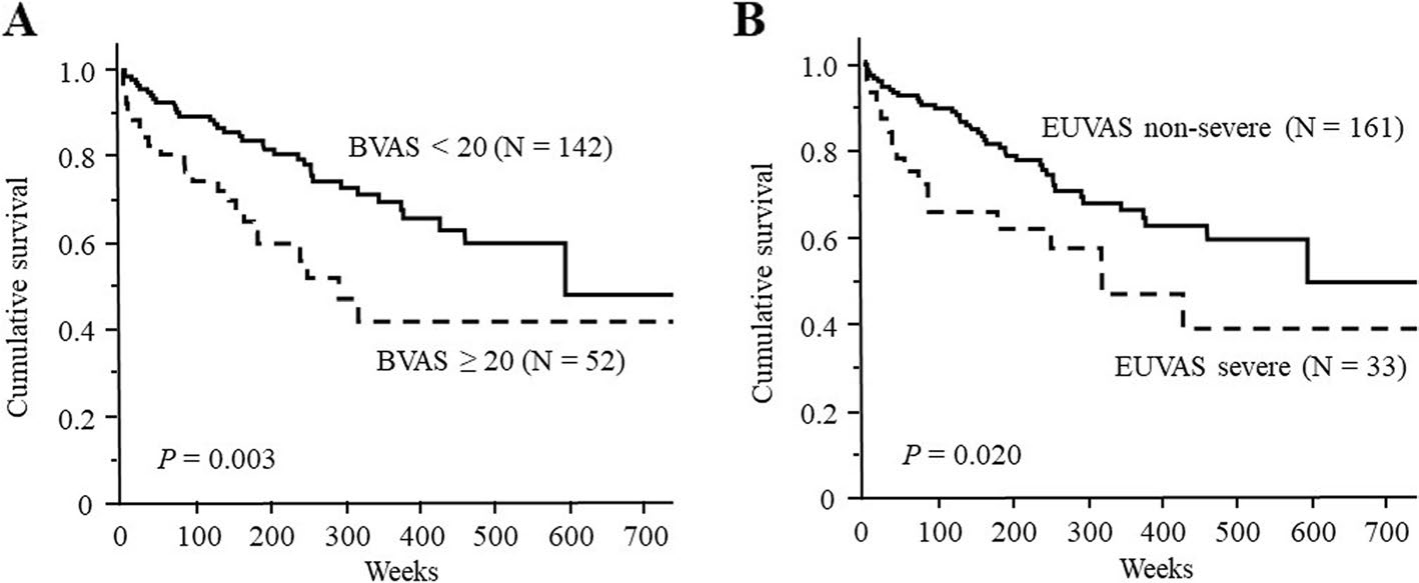
Survival curves of patients with MPA based on BVAS/EUVAS categorization systems. **A** The survival rates over the entire follow-up period were significantly lower in patients with BVAS ≥ 20 than in those with <20 (*P* = 0.003). **B** The survival rates over the entire follow-up period were significantly lower in patients with severe EUVAS than in those with non-severe EUVAS (*P* = 0.020). Survival rates were calculated using the Kaplan–Meier method and compared using the log-rank test

The patients were then divided into two groups based on BVAS at onset (split at the cut-off level), and they were also divided into two groups based on the severe or non-severe EUVAS categorization system; finally, Kaplan–Meier survival curves were plotted for these groups (Fig. 1A, B). The survival rate over the entire follow-up period was significantly lower in patients with BVAS ≥20 than in those with <20 (*P* = 0.003) (Fig. 1A). The survival rate over the entire follow-up period was significantly lower in patients with severe EUVAS designation than in those with the non-severe designation (*P* = 0.020) (Fig. 1B).

### RPM for mortality based on clinical characteristics of MPA

**Fig. 2:**
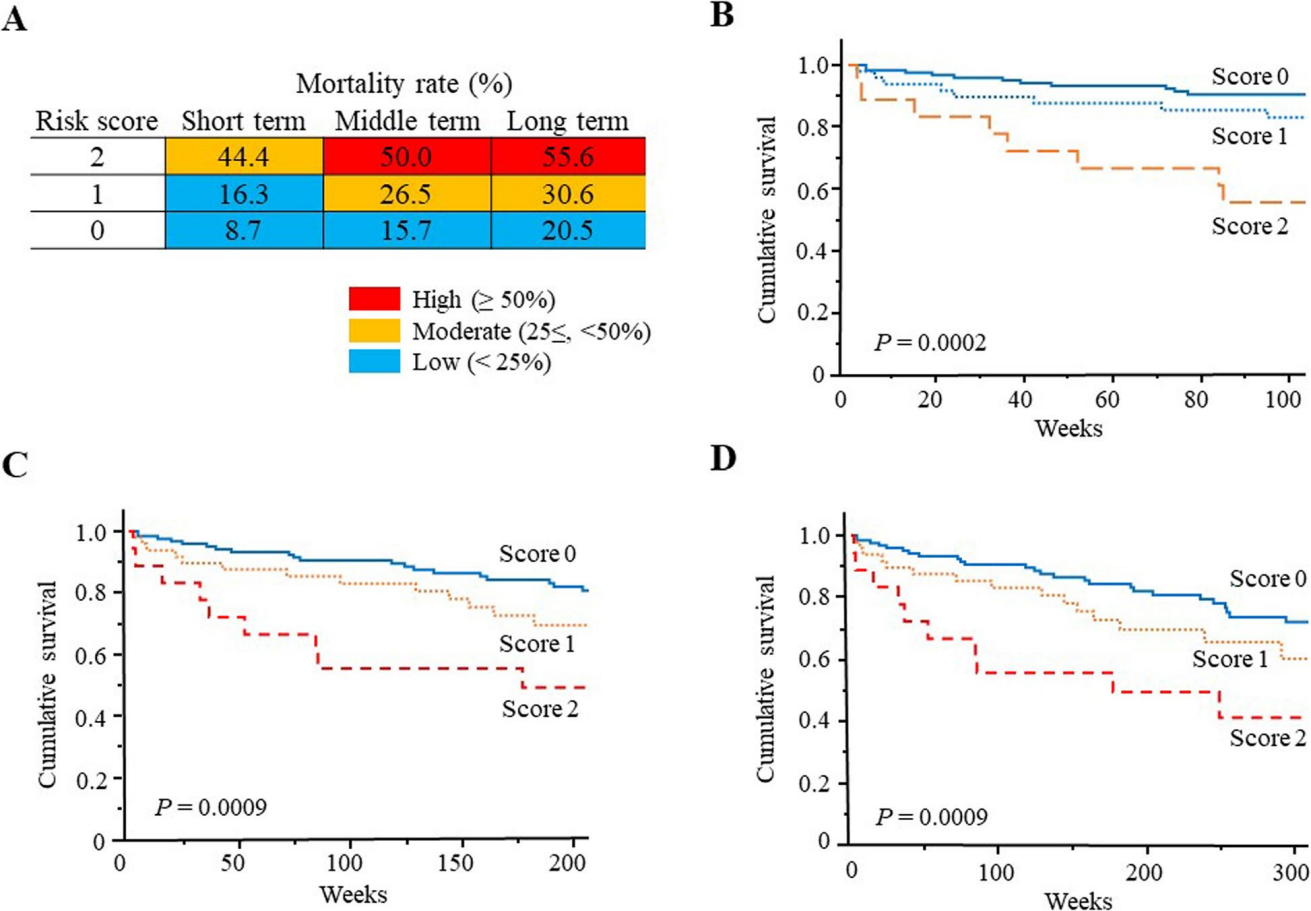
Risk prediction model for mortality in patients with MPA based on BVAS/EUVAS categorization systems. **A** Cumulative mortality rates in patients with MPA, stratified by risk score. The risk score was defined as the number of risk factors (BVAS ≥20 and a severe EUVAS designation). Short-term (2-year; **B**), mid-term (4-year; **C**), and long-term (6-year; **D**) death-free rates were significantly different among patients with MPA stratified by number of risk factors (*P* = 0.0002, 0.0009, and 0.0029, respectively)

Next, we generated a risk predictive model (RPM) for mortality based on combinations of baseline clinical characteristics of the BVAS ≥20 group and the severe EUVAS group. We built a prognostic matrix model based on the BVAS and EUVAS categorization systems (Fig. 2A). This model stratified patients with MPA into low (mortality rate <25%), moderate (mortality rate 25–50%), and high-risk (mortality rate ≥50%) groups, as reported in a previous study [16].

Furthermore, patients were divided into three groups based on the number of risk factors, and Kaplan–Meier survival curves were plotted (Fig. 2B–D). Short-term (2-year), mid-term (4-year), and long-term (6-year) death-free rates were significantly different among patients with MPA stratified by the number of risk factors (*P* = 0.0002, 0.0009, and 0.0029, respectively).

## Discussion

In this multicentre cohort study, 30.9% of the patients with MPA died during the entire follow-up period, and the causes of death were MPA-related vasculitis (18.3%), infections (50.0%), and other causes (31.7%). At baseline, the death group had significantly older age, higher smoking index, lower serum Alb levels, higher serum CRP levels, higher BVAS and FFS, and a more severe EUVAS categorization than the survival group. According to multivariate analyses adjusted for age, two factors were independently associated with death: a high BVAS score and a severe EUVAS designation. The combination of these two poor prognostic factors is useful to predict mortality in patients with MPA.

A systematic review investigating the long-term prognosis of MPA revealed that vasculitis was responsible for mortality in 38–50% of patients, whereas immunosuppressive treatment-related deaths were observed in 17–62% of patients [2]. In a study focusing on the causes of death in Korean patients with AAV, 14 of the 153 patients (9.2%) died during an average follow-up period of 56.9 months (including seven patients with MPA), with infections being the predominant cause of death [5]. This study confirms that infections are the leading cause of death in MPA patients, followed by vasculitis, as shown in earlier findings. Among infection-related mortalities, pulmonary infections were the predominant factor; therefore, employing periodic surveillance measures such as chest imaging, serum β-D-glucan testing, and cytomegalovirus antigen testing for the early detection and treatment of pulmonary infections may represent a crucial strategy for enhancing patient prognoses.

The BVAS is an activity index for AAV, scoring 56 symptoms/signs across nine organ systems encompassing the entire body [12, 17]. BVAS can reportedly predict poor prognosis in patients with MPA [18–20]. A study involving 55 patients with MPA revealed that BVAS was a useful predictor of survival, with adverse outcomes being associated with high BVAS scores, age >60 years, and the presence of interstitial lung disease [19]. Similarly, a study focusing on 73 Japanese patients with MPA reported significantly shorter survival times and higher mortality rates in a group with BVAS scores ≥16 [20]. In line with previous research, our study in Japan found a significant association between BVAS scores of 20 and poor life prognosis in patients with MPA. These findings underscore the importance of early BVAS assessment and appropriate management to optimize clinical outcomes in patients with MPA.

The EUVAS-defined disease severity categories established by EULAR in 2009 serve as a classification system for AAV severity to aid treatment decisions [21]. Regarding the association between the prognosis of patients with MPA and the EUVAS categorization system, a study involving 121 Japanese patients with AAV, including 78 with MPA, revealed that the group with a severe EUVAS designation had significantly lower 2-year survival rates than the generalized group [22]. In the EUVAS categorization system, severe is defined as a condition with renal failure or dysfunction of the vital organs, especially renal lesions, defined as serum Cre ≥ 5.66 mg/dL. We also found a significant relationship between the severe EUVAS categorization and an unfavourable survival prognosis in patients with MPA. Thus, patients with severe disease according to the EUVAS categorization may experience reduced survival rates. Nonetheless, further investigations are needed to elucidate the specific relationship between the EUVAS categorization system and survival in patients with MPA.

The French Vasculitis Study Group Relapse Score, which had been previously introduced for assessing the probability of relapse in patients with MPA and granulomatosis with polyangiitis (GPA), has been reported [23]. Another RPM has also been introduced aimed at predicting cardiovascular events during a 5-year period following the onset of MPA and GPA [24]. In addition to these, Chen et al. proposed a unique RPM in a retrospective single-centre study targeting AAV patients, including 303 patients with MPA; they reported that their RPM demonstrated superior performance compared to FFS and BVAS in predicting life prognosis [25]. In this study, we proposed a simple RPM to predict the prognosis of patients with MPA enrolled in a multicentre cohort of Japanese patients with vasculitis. Our RPM, combining the internationally recognized BVAS and EUVAS categorization systems, demonstrated a high potential for general applicability.

However, several limitations that may have affected the interpretation and generalizability of our findings should be acknowledged. First, as a multicentre study, variations in clinical practices and patient characteristics among the participating centres could have introduced potential bias and confounding factors. Second, the retrospective nature of the study design may have led to missing data and incomplete patient records. We also failed to establish causality between the identified poor prognostic factors and mortality because of the observational nature of our study. Moreover, despite adjusting for age, unaccounted-for confounding factors influencing the observed associations may still exist. Additionally, the cohort study design may limit the establishment of temporal relationships between the risk factors and outcomes. Furthermore, the disease duration from the onset of MPA to the initiation of treatment, for which the data were not available, should be considered for future investigations, as it holds the potential to impact patient prognosis. Finally, this study focused on Japanese patients with MPA, which limits the generalizability of our findings to other populations.

## Conclusions

Our study provides valuable insights into the prognostic factors for MPA mortality. Recognizing the study limitations, further large-scale, diverse, population-based studies are needed to validate and expand our findings. Our RPM offers a significant contribution to vasculitis research with potential clinical implications for risk assessment and patient management. Further validation and prospective studies are necessary to assess its robustness and applicability in diverse MPA populations. Overall, our research advances the understanding of MPA prognosis and opens avenues for future investigation and clinical applications.

### Supplementary Information

**Additional file 1.** Details of cause of death.

**Additional file 2.** ROC curves of BVAS to differentiate demised patients in MPA. ROC: receiver operating characteristic; BVAS: Birmingham Vasculitis Activity Score; AUC: area under the curve.

## Data Availability

The datasets used and/or analysed during the current study are available from the corresponding author upon reasonable request.

## Abbreviations

MPA: Microscopic polyangiitis
ANCA: Anti-neutrophil cytoplasmic autoantibody
AAV: ANCA-associated vasculitis
REVEAL: Registry of Vasculitis Patients to Establish the REAL World Evidence
WBC: White blood cell
Hb: Haemoglobin
Alb: Albumin
Cre: Creatinine
CRP: C-reactive protein
MPO-ANCA: Myeloperoxidase-ANCA
PR3-ANCA: Proteinase 3-ANCA
BVAS: Birmingham Vasculitis Activity Score
EUVAS: European Vasculitis Study Group
FFS: Five-Factor Score
PDN: Prednisolone
MPDN: Methylprednisolone
IVCY: Intravenous cyclophosphamide
RTX: Rituximab
IVIG: Intravenous immunoglobulin
AZA: Azathioprine
MMF: Mycophenolate mofetil
HR: Hazard ratio
CI: Confidence interval
ROC: Receiver operating characteristic
RPM: Risk predictive model
