# Supplementary material for: Risk prediction model for mortality in microscopic polyangiitis: multicentre REVEAL cohort study
Source: Arthritis Res Ther. 2023 Nov 20;25:223. doi: 10.1186/s13075-023-03210-8 (PMC10658814; doi:10.1186/s13075-023-03210-8)
Supplement: Supplementary file 1 — Additional file 1. Details of cause of death. [file 13075_2023_3210_MOESM1_ESM.docx]

**Additional file 1.**

| Details of mortality-causes | |
| --- | --- |
| Cause of death, n (%) | Details, n (%) |
| MPA-related vasculitis, 11 (18.3) | Diffuse alveolar hemorrhage, 5 (45.5) |
|  | Acute exacerbation of interstitial lung disease, 3 (27.3) |
|  | Gastrointestinal bleeding, 1 (9.1) |
|  | Cerebral hemorrhage, 1 (9.1) |
|  | Systemic vasculitis, 1 (9.1) |
| Infections, 30 (50.0) | Bacterial pneumonia, 19 (63.3) |
|  | *Pneumocystis jirovecci* pneumonia, 1 (3.3) |
|  | Cytomegalovirus pneumonia, 1 (3.3) |
|  | Pulmonary tuberculosis, 1 (3.3) |
|  | Aspergillus pneumonia, 1 (%) |
|  | Sepsis, 4 (13.3) |
|  | Infective endocarditis, 2 (6.7) |
|  | Pyelonephritis, 1 (3.3) |
|  | Cholangitis, 1 (%) |
| Others, 19 (31.7) | Malignancy, 2 (10.5) |
|  | Heart failure, 2 (10.5) |
|  | Mediastinal hematoma, 1 (5.3) |
|  | Aortic valve stenosis, 1 (5.3) |
|  | Liver failure, I (5.3) |
|  | Acute abdomen, 1 (5.3) |
|  | Acute respiratory failure, 1 (5.3) |
|  | Cerebral infarction, 1 (5.3) |
|  | Senile decay, 1 (5.3) |
|  | Unknown, 8 (42.1) |
