# Supplementary material for: Risk prediction model for mortality in microscopic polyangiitis: multicentre REVEAL cohort study
Source: Arthritis Res Ther. 2023 Nov 20;25:223. doi: 10.1186/s13075-023-03210-8 (PMC10658814; doi:10.1186/s13075-023-03210-8)
Supplement: Supplementary file 2 — Additional file 2. ROC curves of BVAS to differentiate demised patients in MPA. ROC: receiver operating characteristic; BVAS: Birmingham Vasculitis Activity Score; AUC: area under the curve. [file 13075_2023_3210_MOESM2_ESM.docx]

**Additional file 2.** **ROC curves of BVAS to differentiate demised patients in MPA.**


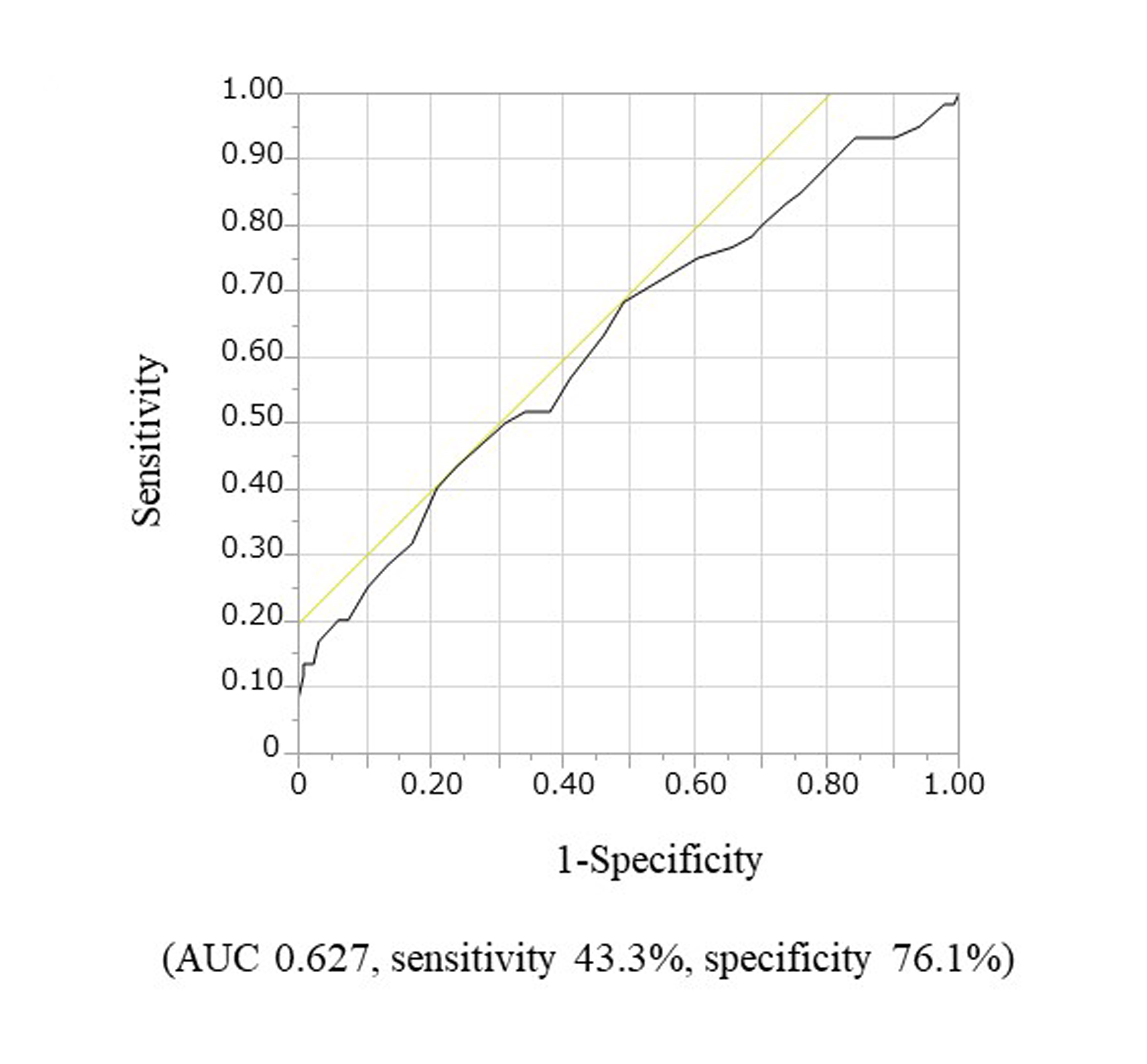


ROC: receiver operating characteristic; BVAS: Birmingham Vasculitis Activity Score; AUC: area under the curve.
